# Decreased Thymic Output Contributes to Immune Defects in Septic Patients

**DOI:** 10.3390/jcm9092695

**Published:** 2020-08-20

**Authors:** Natascha Sommer, Steffen Noack, Andreas Hecker, Holger Hackstein, Gregor Bein, Norbert Weissmann, Werner Seeger, Konstantin Mayer, Matthias Hecker

**Affiliations:** 1 Excellence Cluster Cardio-Pulmonary Institute (CPI), University of Giessen and Marburg Lung Center (UGMLC), Member of the German Center for Lung Research (DZL), Justus-Liebig-University of Giessen, 35392 Giessen, Germany; Natascha.Sommer@innere.med.uni-giessen.de (N.S.); Steffen_Noack@gmx.de (S.N.); Norbert.Weissmann@innere.med.uni-giessen.de (N.W.); Werner.Seeger@innere.med.uni-giessen.de (W.S.); Konstantin.Mayer@innere.med.uni-giessen.de (K.M.); 2 Department of General and Thoracic Surgery, Justus-Liebig-University of Giessen, 35392 Giessen, Germany; Andreas.Hecker@chiru.med.uni-giessen.de; 3 Department of Transfusion Medicine and Hemostaseology, University Hospital Erlangen, 91054 Erlangen, Germany; Holger.Hackstein@uk-erlangen.de; 4 Institute for Clinical Immunology and Transfusion Medicine, Justus-Liebig-University, 35392 Giessen, Germany; Gregor.Bein@immunologie.med.uni-giessen.de; 5 Department of Pulmonary and Sleep Medicine, ViDia hospitals, 76137 Karlsruhe, Germany

**Keywords:** sepsis, thymus, lymphopenia, inflammation

## Abstract

Background: Prolonged immunosuppression and hypoinflammation, termed compensatory anti-inflammatory response syndrome (CARS), contribute to high morbidity and mortality in the late phase of sepsis. Although apoptosis is a well-known cause of lymphopenia in sepsis, the contribution of thymic output to immune alterations in sepsis and potential compensatory mechanisms are largely unknown. Methods: We investigate the release of CD4+ T cells from the thymus and their peripheral proliferation by evaluating T-cell receptor excision circles (TREC) and the expression of CD31 as markers for recent thymic emigrants (RTE) and their proliferative offspring in septic patients with relevant lymphopenia in the CARS phase. Moreover, we determine the aging of T cells by measuring telomere characteristics. Results: In septic patients, we found decreased CD4+ T-helper cell numbers, while CD8+ T cell numbers were unchanged. As a possible cause, we detected increased apoptosis of CD4+ T-helper cells and decreased levels of IL-7, which promotes the maturation of T cells in the thymus. Accordingly, the relative number of mature CD4+ T cells, TREC-containing CD4+ T cells, and CD31+ RTEs (characteristic of thymic output) was decreased, while the relative number of CD31-T cells (peripherally expanded naïve T cells) was increased. Furthermore, the telomere length decreased, although telomerase activity and markers for the shelterin complex were increased specifically in CD4+ but not in CD8+ T cells. Conclusion: We thus conclude that, in addition to T-cell apoptosis, decreased thymic output and increased aging of CD4+ T cells may contribute to lymphopenia and immunosuppression in sepsis. Increased proliferation of peripheral T cells cannot compensate for these effects.

## 1. Introduction

Sepsis and septic shock are leading causes of morbidity and mortality in intensive care units worldwide; their incidence is increasing, especially among elderly patients [1,2,3]. The pathogenesis of sepsis is complex and not yet fully elucidated. Most recent understandings of these conditions are based on a bi-phasic course of the disease, starting with hyperinflammation (often referred to as a “cytokine storm”) as a direct reaction to systemic pathogenic invasion, a phenomenon referred to as SIRS (systemic inflammatory response syndrome). SIRS is characterized by the elevated presence in systemic circulation of inflammatory mediators that promote the immune response (e.g., leukocyte recruitment) but also potentially cause cardiovascular dysfunction, activation of coagulation, or edema formation [4]. Patients surviving this early phase of sepsis may enter a state of prolonged immunosuppression and hypoinflammation, termed CARS (compensatory anti-inflammatory response syndrome), which may have the physiological function of restoring immune homeostasis after an inflammatory state. Several studies have indicated that CARS is not merely the state following SIRS, but that both phases occur independently in sepsis [5,6,7]. Moreover, the late phase of sepsis, in particular, accounts for the majority of deaths due to the interplay of an ongoing infection facing a suppressed immune system [4,8]. Several mechanisms have been proposed to explain the observed immunodepression that affects both the adaptive and innate immune systems. One hallmark in the pathogenesis of particularly the late phase of sepsis is the appearance of apoptotic immune cells, and profound depletion of immune cells [9,10]. In this context, significant apoptosis of T and B cells translates clinically into a persistent lymphopenia in the peripheral blood, often associated with a poor prognosis [11,12]. The role of potential compensatory mechanisms to reestablish T-cell homeostasis, such as clonal expansion of surviving cells or alterations in thymic function, is currently widely unaddressed in septic shock patients. In this study, we aim to investigate the replicative history, clonal expansion, and age of CD4+ and CD8+ T lymphocytes in septic shock patients. We use prospective measurements of T-cell receptor excision circles (TREC) and recent thymic emigrants (RTEs) to assess human thymic output, focusing on patients with relevant lymphopenia in the CARS phase. Furthermore, we investigate telomere length and telomerase activity in CD4+ and CD8+ lymphocytes to evaluate whether excessive proliferative pressure or inadequate telomeric maintenance may account for the observed lymphopenia in septic shock patients.

## 2. Materials and Methods

### 2.1. Study Population

This single-center observational study was performed in the medical intensive care unit of the University Hospital of Giessen. We enrolled fifteen patients with severe sepsis, diagnosed according to the criteria from the “Surviving Sepsis Campaign” [13] (mean age (±SD): 67.1 (12); 73% male). All patients had respiratory failure requiring mechanical ventilation on enrollment. Patients with active malignancy or HIV infection and those taking immunosuppressive or immunomodulatory treatment were excluded. This observational study was approved by the local ethics committee (Ethikkommission des Fachbereichs Medizin, Justus-Liebig University Giessen, AZ107/08); written informed consent was obtained from the patients’ legal representatives, as all patients enrolled were incapable of providing consent due to their critical illness. Clinical data on the septic patients were monitored and documented daily. Age- and sex-matched healthy blood donors were chosen as controls (mean age (±SD): 65.4 (15.3); 73% male).

### 2.2. Sample Collection and Processing

Per clinical routine, blood was collected via a central venous catheter or arterial line. Residual blood remaining from analysis of routine parameters was collected and subjected to further experimental procedures, being optimized for the use of small cell counts. For analysis of cytokine concentrations, plasma was collected and stored at −80 °C.

### 2.3. CD4+ and CD8+ T Cell Isolation

CD4+ and CD8+ T cells were positively selected with magnetic beads and subsequently sorted with autoMACS (Miltenyi Biotec, Bergisch Gladbach, Germany). Reagents were used according to the manufacturer’s instructions. The purity of positively selected T cells was >98%, as determined by flow cytometry.

### 2.4. Flow Cytometry and Staining Procedure

We performed cellular phenotyping on a FACS Canto II flow cytometer (Becton Dickinson, San Jose, CA, USA). To identify CD4 subsets, we used the following fluorochrome-labeled antibodies for surface staining, according to the manufacturer’s instructions: CD45RA-Pacific Blue and CD31-APC (Biolegend, San Diego, CA, USA), and CD4-APC-H7 and CD45RO-PE (BD Pharmingen, San Diego, CA, USA). Isotype-matched control antibodies were ordered from the same companies. After performing an automated cell count, we incubated 50 µL of blood with erythrocyte-lysing buffer (BD-Pharm Lyse, Frankin Lakes, NJ, USA) at room temperature, centrifuged the solution, and discarded the supernatant. The staining time for surface antibodies or isotypes was 30 min, followed by washing with staining buffer (1 × PBS/5% FBC, both reagents from PAA, Coelbe, Germany). Staining was performed on ice.

### 2.5. Protein Miniarrays

To analyze apoptosis- or cell stress-related proteins, we subjected cell lysates (CD4 and CD8 lymphocytes) from controls and sepsis patients to protein miniarrays (Human Apoptosis Array, Human Cell Stress Array, R&D Systems, Wiesbaden, Germany), performed according to the manufacturer’s instructions. Due to sample limitation, we performed this experiment one time with pooled cell lysates to confirm previously described regulation of apoptotic pathways [14]. To evaluate changes in spot intensity, blots were visually evaluated by three independent investigators. Spots marked in the manuscript are those which all three investigators described as being changed [15].

### 2.6. Determination of IL-7 and IL-15

IL-7 and IL-15 serum levels were determined with a commercially available ELISA kit (R&D Systems, Wiesbaden, Germany), according to the manufacturer’s instructions.

### 2.7. Analysis of Telomerase Activity

CD4+ and CD8+ T cells were sorted with magnetic beads, as described above, to achieve equal numbers of both subsets. Telomerase activity was measured using a Telomerase PCR ELISA Kit (Roche Diagnostics, Basel, Switzerland), based on a Telomeric Repeat Amplification Protocol (TRAP) assay. In the first step, telomerase-mediated elongation products were amplified by PCR, followed by immobilization of the biotinylated PCR product onto a streptavidin-coated microtiter plate. Detection was subsequently performed with the ELISA technique, using an antibody against digoxigenin conjugated to peroxidase. Finally, the probe was visualized by addition of peroxidase substrate (3,3′,5,5’-tetramethyl benzidine) to form a colored reaction product, whose absorbance was measured at 450 nm (A_450 nm_). Telomerase activity is reported as A_450 nm_, measured against the blank (A_690 nm_).

### 2.8. Telomere Length Analysis

We assessed telomere length by real-time quantitative RT-PCR, based on the T/S method by Cawthon [16]. The T/S ratio was generated by determination of the mean relative quantity (nanogram equivalent) of sequence for telomere (T) and a single-copy gene (s). Thus, the T/S ratio, given in arbitrary units, measures the quantity of telomeric DNA per quantity of single-copy DNA sequence; it reflects the average telomere length [16]. In brief, DNA was extracted from CD4+ and CD8+ T cells as previously described. For each sample tested, two successive quantitative PCR reactions were performed using 30 ng each of the respective DNA sample and a reference series of diluted standard DNA (2.5–80 ng). The first reaction amplified a telomere sequence (T) using the following primers: Tel_forward_ 5′-GGTTTTTGAGGGTGAGGGTGAGGGTGAGGGTGAGGGT-3′ and Tel_reverese_ 5′-TCCCGACTATCCCTATCCCTATCCCTATCCCTATCCCTA-3′. The second PCR reaction amplified part of the single-copy gene 36B4 (S), encoding the ribosomal phosphoprotein P0, with the following primers: 36B4_forward_ 5′-CAGCAAGTGGGAAGGTGTAATCC-3′ and 36B4_reverse_ 5′-CCCATTCTATCATCAACGGGTACAA-3′. For the PCR reaction, the thermal cycling profile began with a 10 min activation at 95 °C, followed by 35 cycles at 95 °C for 15 s and 54 °C for 2 min (for T; for S, 1 min) [16].

### 2.9. T-Cell Receptor Excision Circle (TREC) Analysis

To estimate the rate of thymic CD4+ lymphocyte export, we performed an analysis of T-cell receptor excision circles (TREC)—extrachromosomal DNA byproducts of T-cell receptor gene rearrangement. For the experimental procedure, DNA was extracted from isolated CD4+ T cells using the QIAamp DNA Mini Kit (QIAGEN, Chartsworth, CA, USA) according to the manufacturer’s instructions. The quantification of TRECs was performed by SYBR Green quantitative RT-PCR, using the protocol described by Ponchel and colleagues [17]. For this method, the number of cells in the reaction is estimated using *GAPDH* copy number and compared to the TREC copy numbers assessed by the PCR protocol. *GAPDH* and a TREC assay are run in parallel to normalize the number of TRECs to the number of CD4+T-cells in the sample giving the number of cells containing a TREC as a percentage CD4 containing TREC.

### 2.10. RT-PCR of Telomerase- and Shelterin-Associated Genes

We performed the extraction of RNA from isolated CD4+ and CD8+ T cells, the generation of cDNA, and gene expression analysis by quantitative real-time RT-PCR, as described previously [15,18,19]. The following primers were used for amplification of telomerase- and shelterin-associated genes: TERC (for: 5′-GAAGAGGAACGGAGCGAGTC, rev: 5′-AAAAAGCGGAAGACG GGAG), TERT (for: 5′-CGGCTTTTGTTCAGATGCC; rev: 5′-AGCACACATGCGTGA AACCT), TRF1 (for: 5′-GCTGTTTGTATGGAAAATGGC, rev: 5′-CCGCTGCCTTCAT TAGAAAG), TRF2 (for: 5′-GACCTTCCAGCAGAAGATGC; rev: 5′-GTTGGAGGATTCC GTAGCTG), POT1 (for: 5′-TGGGTATTGTACCCCTCCAA; rev: 5′-GATGAAGCATTCC AACCACGG), and RAP1 (for: 5′-CGGGGAACCACAGAATAAGA; rev: 5′-CTCAGGTGTGGGTGGATCAT). Normalization of the results was performed using the housekeeping gene GAPDH (for: 5′-AACAGCGACACCCATCCTC, rev: 5′-CATACCAGGAAATGAGCTTGACAA).

### 2.11. Statistics

Data are presented as the means ± SEMs. Student´s T-test was performed for comparison of two groups. Probability (*p*) values < 0.05 were considered statistically significant. Analyses were carried out using GraphPad Prism 6 (GraphPad Software Inc., San Diego, CA, USA) for Windows.

## 3. Results

### 3.1. Lymphopenia, Apoptosis, and Proliferation in Patients with Septic Shock

**Figure 1:**
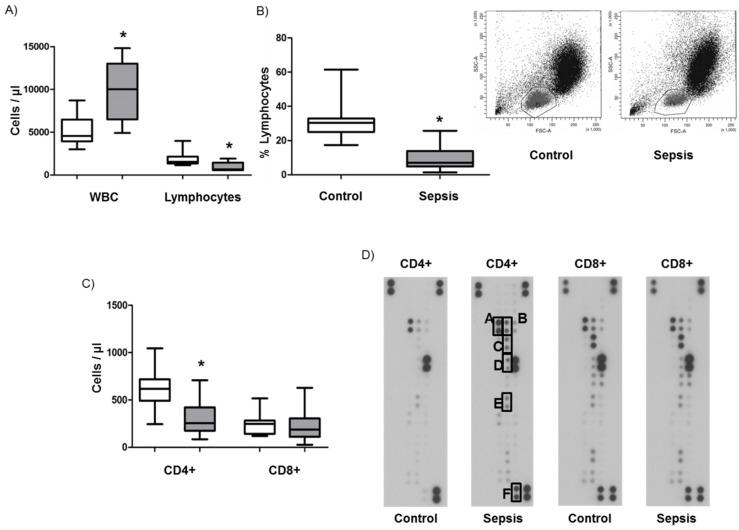

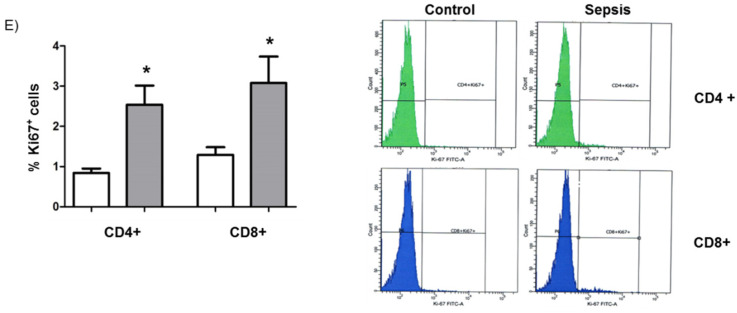
Lymphopenia, apoptosis, and proliferation in sepsis patients and control patients. (**A**) Cell counts of white blood cells (WBC) and lymphocytes in healthy controls (white bars) and sepsis patients (grey bars). (**B**) Percentage of lymphocytes in the peripheral blood of controls and sepsis patients. (**C**) Absolute numbers of CD4+ and CD8+ lymphocytes in the blood of controls (white bars) and sepsis patients (grey bars) assessed by magnetic cell sorting and counting. (**D**) Apoptosis protein array of CD4+ and CD8+ cells (homogenate) derived from controls and sepsis patients. The letters A-F indicate regulated proteins (A: p27/Kip1; B: Fas-associated protein with death domain (FADD); C: Fas; D: Hypoxia-inducible factor (HIF)-1α; E: Heme oxygenase 2 (HMOX); F: Livin). The assay was performed one time with pooled samples. (**E**) Proliferation of CD4+ and CD8+ lymphocytes assessed by Ki67 staining (white bars: control; grey bars: sepsis) with representative flow cytometric charts. Data are displayed as a box-and-whisker plot representing the 25th and 75th percentiles (bottom and top of the box), the median (band inside the box), and the minimum and maximum of all data (ends of the whiskers) for (**A**–**C**). Data are displayed as mean ± SEM for (**E**) Asterisks (*) denote statistical significance, with *p* < 0.05.

Septic shock patients enrolled in the study showed significantly elevated numbers of white blood cells (Figure 1A, WBC, 9971/µL versus 5118/µL for controls) and a significantly reduced absolute and relative number of total lymphocytes in their peripheral blood, as compared to age-and sex-matched healthy controls (Figure 1A–C, 30.4% versus 9.3%; * *p* < 0.05). Magnetic cell sorting and subsequent counting of their phenotypes revealed that, in septic patients, the absolute number of CD4+ T cells was significantly decreased (Figure 1C, 321 CD4+ T cells/µL blood versus 608 CD4+ T cells/µL for healthy controls; * *p* < 0.05). In contrast, CD8+ T cells showed no significant difference between the two groups (Figure 1C).

To confirm the previously described role of apoptosis as a potential explanation for the observed CD4+ dominated lymphopenia, we performed a protein array of the most common apoptosis-related molecules of isolated CD4+ and CD8+ T cells (Figure 1D). As expected, we detected an upregulation of one major pro-apoptotic pathway involving Fas (C)/FADD(B) signaling, two other proteins which can promote apoptosis (p27/Kip1 (A); HIF-1α (D)), and two anti-apoptotic molecules (HMOX2 (E); Livin (F)) in CD4+ T cells derived from patients with septic shock compared to controls. Interestingly, CD8+ T cells from controls compared to samples from sepsis patients displayed even less apoptosis-related proteins. To investigate the proliferation rates of CD4+ and CD8+ T cells as a potential explanation for the differential lymphocyte counts in septic shock, we stained cells for Ki67. In patients and controls, the proliferation rates were very low, varying from 1–3% of Ki67-positive cells in both cell types. Nevertheless, CD4+ T cells from septic shock patients displayed a significantly higher proliferation rate than those from healthy controls (Figure 1E).

### 3.2. Cytokine Levels in Septic Shock Patients

**Figure 2:**
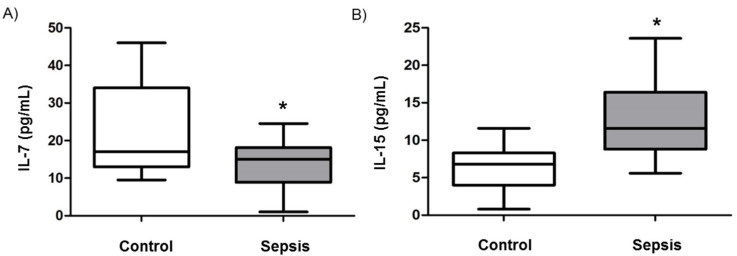
Cytokine levels in septic patients. Serum concentrations of Interleukin-7 (**A**) and Interleukin-15 (**B**) were assessed in healthy controls (white bars) and sepsis patients (grey bars). Cytokine concentrations are displayed as a box-and-whisker plot representing the 25th and 75th percentiles (bottom and top of the box), the median (band inside the box), and the minimum and maximum of all data (ends of the whiskers). Asterisks (*) denote statistical significance, with *p* < 0.05.

To further investigate whether the cytokine milieu in sepsis might affect T-cell homeostasis and thus be involved in the pathogenesis of septic lymphopenia, we measured the serum concentration of Interleukin-7 (IL-7), a key mediator of T-cell homeostasis and proliferation which was decreased in septic patients, improved survival in animal models of sepsis and increased lymphocyte numbers in septic patients [20]. In line with the results on CD4+ T cell numbers, the levels of IL-7, which are required for T cell survival, expansion, and development of mature T cells in the thymus, were downregulated (Figure 2A). In contrast, IL-15, which was increased or unchanged in serum of septic patients [20,21]), promoted sepsis in animal models and plays an important role specifically in CD8+ T cell survival [22], was upregulated (Figure 2B).

### 3.3. Role of Recent Thymic Emigrants and Thymic Dysfunction in Septic Lymphopenia

Unlike in early childhood, when thymic export of lymphocytes is primarily responsible for the quality and quantity of the naïve T-cell pool, T-cell homeostasis and diversity in adults is determined primarily by post-thymic expansion of naïve recent thymic emigrants (RTEs). To investigate the hypothesis that a loss of RTEs—associated with a decline in thymic activity and output—plays a role in sepsis-induced lymphopenia, we assessed RTE content with two experimental markers that characterize RTEs and their peripheral proliferative offspring.

**Figure 3:**
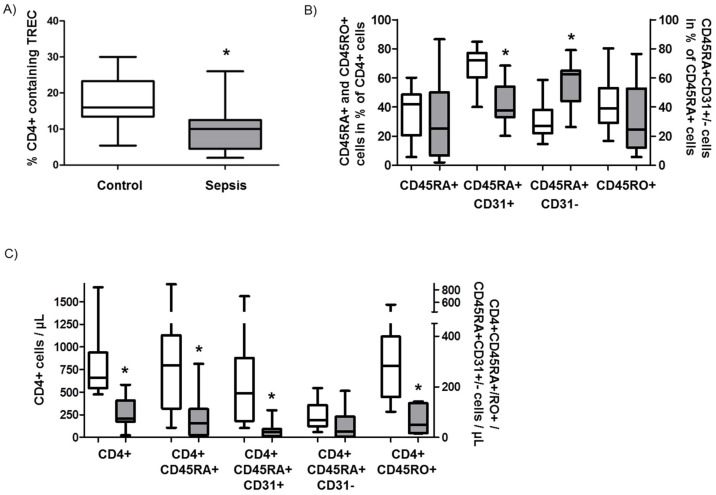

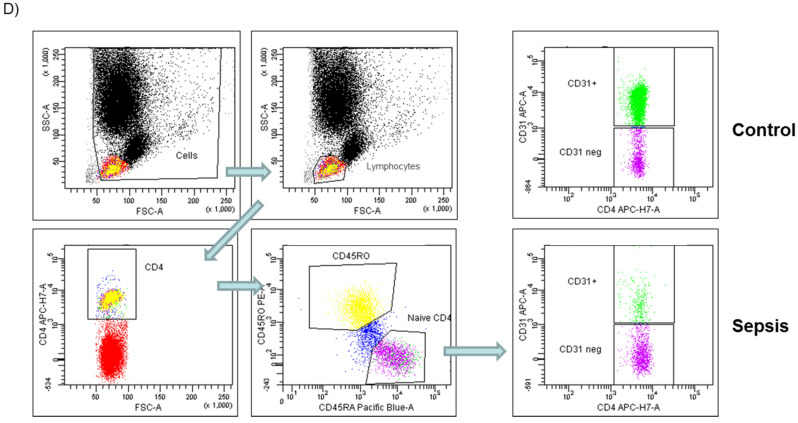
Recent thymic emigrants (RTE) in sepsis patients. (**A**) Determination of the content of TRECs (T-cell receptor excision circle) in CD4+ T cells. (**B**) Assessment of naïve T cells (CD4+CD45RA+), memory T cells (CD4+CD45RO+), RTEs (CD31+CD4+CD45RA+) and peripheral naïve T cells (CD31CD4+CD45RA-) in the blood of sepsis patients and controls (displayed as relative percentage of CD4+ cells). (**C**) Absolute numbers of naïve T cells (CD4+CD45RA+), memory T cells (CD4+CD45RO+), RTEs (CD31+CD4+CD45RA+) and peripheral naïve T cells (CD31CD4+CD45RA-) in the blood of sepsis patients and controls. (**D**) Representative flow cytometry stainings for above-mentioned experiments. Data are displayed as a box-and-whisker plot representing the 25th and 75th percentiles (bottom and top of the box), the median (band inside the box), and the minimum and maximum of all data (ends of the whiskers). Asterisks (*) denote statistical significance, with *p* < 0.05.

First, we performed a PCR-based assay to assess T-cell maturation by determining the content of TRECs (T-cell receptor excision circle) in CD4+ T cells. TRECs are excision byproducts generated during T-cell development in the thymus; they are enriched in RTEs but diluted during peripheral proliferation of naïve T cells. The TREC content in the CD4+ T cells of patients with septic shock was significantly reduced compared to age- and sex-matched controls (Figure 3A), indicating decreased RTEs in sepsis.

Secondly, we determined the relative and absolute number of RTEs and their proliferative offspring by flow cytometric staining for CD31+CD4+CD45RA+ and CD31-CD4+CD45RA+ T-cells, respectively, in the peripheral blood of sepsis patients and controls. We found that the relative percentage of naïve T cells (CD4+CD45RA+) was similar in sepsis and controls (Figure 3B). However, the relative number of RTEs (CD31+CD4+CD45RA+) was significantly lower in septic shock patients than in controls (* *p* < 0.05), whereas the relative number of peripherally post-thymically expanded naïve T cells (CD31-CD4+CD45RA+) was higher in septic shock patients (Figure 3B). The content of memory T cells (CD4+CD45RO+) did not differ between the two groups (Figure 3B).

**Figure 4:**
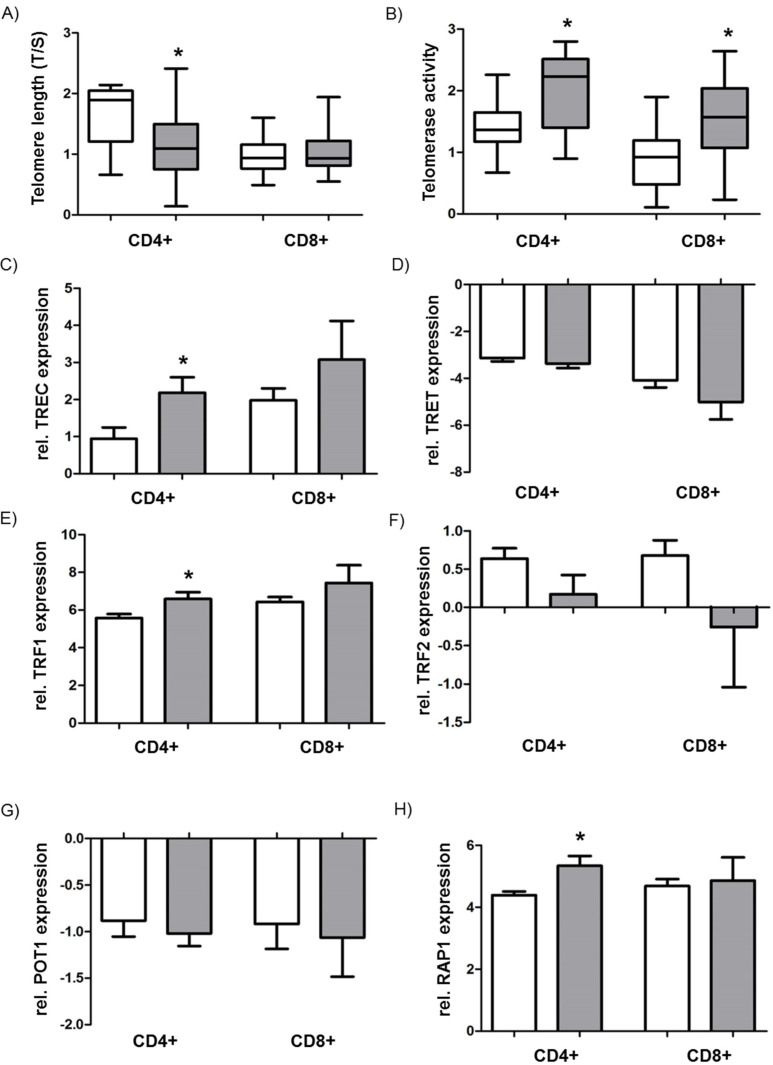
Analysis of telomere length, telomerase activity, and the shelterin complex. (**A**) Assessment of telomere length by T/S ratio in CD4+ and CD8+ T cells of septic patients (grey bar) and controls (white bar). (**B**) Telomerase activity determined by telomeric repeat amplification protocol (TRAP) assay in CD4+ and CD8+ T cells of septic patients (grey bar) and controls (white bar). (**C**) mRNA expression of TRET (telomerase reverse transcriptase) and (**D**) TERC (telomerase RNA component) in CD4+ and CD8+ T cells of septic patients (grey bar) and controls (white bar). mRNA expression of shelterin complex components TRF1 (Telomeric Repeat Factor 1) (**E**), TRF2 (**F**), POT1 (Protection of Telomeres 1) (**G**), and RAP1 (Repressor/Activator protein 1) (**H**) in CD4+ and CD8+ T cells of septic patients (grey bar) and controls (white bar). mRNA expression is provided as relative expression compared to the housekeeping gene GAPDH. Data are displayed as a box-and-whisker plot representing the 25th and 75th percentiles (bottom and top of the box), the median (band inside the box), and the minimum and maximum of all data (ends of the whiskers) for (**A,B**) Data are displayed as mean ± SEM for (**C**–**H**). Asterisks (*) denote statistical significance, with *p* < 0.05.

To address the fact that the total number of lymphocytes was dramatically reduced in sepsis, the absolute cell counts for the above-mentioned results were subsequently calculated. Both the absolute numbers of naïve T cells (CD4+CD45RA+; 82/µL (sepsis) versus 316/µL (control); * *p* < 0.05) and RTEs (CD31+CD4+CD45RA+; 29/µL (sepsis) versus 220/µL (control); *p* < 0.05) were significantly lower in patients with septic shock (Figure 4C). The concentration of peripherally post-thymically expanded naïve T cells (CD31-CD4+CD45RA+) did not differ significantly between sepsis patients (53/µL) and controls (93/µL). Furthermore, the absolute numbers of memory T cells were significantly lower in septic shock patients than in controls (66/µL versus 302/µL; * *p* < 0.05) (Figure 3C). Representative flow cytometry stainings are provided in Figure 3D.

### 3.4. Analysis of Telomere Length, Telomerase Activity, and the Shelterin Complex

To gain further insight into T-cell dysfunction induced by replicative senescence of the CD4+/CD8+ T cells in the peripheral blood of lymphopenic patients with sepsis, we performed telomere length measurements of separated CD4+ and CD8+ T cells, applying the T/S method. We found that the T/S ratio (reflecting telomere length) in CD4+ T cells of septic shock patients was significantly reduced compared to controls (Figure 4A, 1.17 (sepsis) versus 1.69 (control); * *p* < 0.05). The telomere lengths of chromosomes in CD8+ T cells showed no relevant difference between sepsis patients and their respective controls (1.07 versus 0.97 for controls) (Figure 4A).

In addition, the activity of the enzyme telomerase, responsible for maintaining and regulating telomere length, was analyzed within separated CD4+ and CD8+ T cells using the modified TRAP assay. The results indicate that the CD4+ T cells of septic patients showed significantly higher telomerase activity than those of healthy controls (Figure 4B, * *p* < 0.05). A similar pattern was observed for CD8+ T cells, which also displayed elevated telomerase activity in patients with septic shock compared to controls (Figure 4B, * *p* < 0.05). Using quantitative RT-PCR, we next elucidated the expression of the two key subunits of the telomerase complex: Telomerase reverse transcriptase (TRET) and telomerase RNA component (TERC). The expression of catalytic TERT remained unchanged across the cell types and groups, whereas TERC expression was significantly upregulated in the CD4+ T cells of sepsis patients (Figure 4C,D, * *p* < 0.05).

Finally, we studied the components of the shelterin complex (TRF1, TRF2, POT1, RAP1), which is located at the very end of the telomere to stabilize its structure and integrity. CD4+ T cells derived from sepsis patients exhibited significantly increased expression of TRF1 and RAP1 compared to control samples (Figure 4E,F, * *p* < 0.05). TRF2 and POT1 expression did not differ among the indicated groups and settings (Figure 4G,H).

## 4. Discussion

In this study, we investigate the role of thymic dysfunction in the pathogenesis of severe septic lymphopenia and immunosuppression. We address T-cell homeostasis, which is achieved through a balance of apoptosis, thymic output, and peripheral proliferation. This study demonstrates for the first time that decreased CD4+ T cell numbers in septic patients are associated not only with increased apoptosis but also with thymic dysfunction—characterized by decreased CD4+ T cell output, which is counteracted by increased peripheral proliferation of CD4+ T cells. Although telomerase was upregulated as a protective mechanism against replicative senescence, telomere length in CD4+ T cells was decreased, suggesting that inflammation may promote T-cell senescence and, thereby, possibly contribute to T-cell dysfunction in septic patients. In contrast, CD8+ T cell numbers, apoptosis, and telomere length were not significantly altered in septic patients.

As expected, our study found increased WBC numbers in septic patients compared to healthy controls. Increased WBC numbers and the presence of immature cells in the peripheral circulation are important determinants of an infection. However, in sepsis WBC numbers also may be in the normal range and the ratio of neutrophils to lymphocytes can be used as a more valuable parameter for diagnosis of sepsis [23]. Our findings are in line with previous investigations that have shown a persistent decrease of CD4+ T cell numbers—but not CD8+ T cells—in septic patients [24]. In this investigation a decrease from normal values of CD4+, CD8+, and total T lymphocytes from peripheral blood of septic patients was observed, but the decline persisted only for CD4+ T lymphocytes for three days. Thus, the specific decrease of CD4+ T cells that we observed in our study (with blood drawn 48 to 72 h after diagnosis of sepsis) may also relate to an impaired recovery of CD4+ T cells and not a selective decrease. However, currently the reason for the different behavior of CD4+ and CD8+ T cells is unknown. Several reasons may account for the decreased CD4+ but not CD8+ T cell number such as altered apoptosis including activation-induced cell death and re-distribution between lymphatic tissue and the periphery, which cannot be compensated by increased proliferation. Along these lines, we observed increased expression of pro-apoptotic markers in CD4+ T cells derived from our cohort of septic shock patients, in particular Fas/FADD which is a major component of the extrinsic pathway of apoptosis, suggesting that increased apoptosis contributes to decreased numbers of CD4+ T cells, as described previously [14]. This finding aligns with previous reports describing caspase-3-mediated apoptosis of lymphocytes in sepsis [5,9]. Because T cell numbers may be affected not only by apoptosis of mature CD4+ T cells but also by replenishment of the CD4+ T cell pool by the thymus, we aimed to measure CD4+ T cell output from the thymus. After localization of bone marrow-derived T cell progenitors within the thymus, these thymocytes undergo a series of maturation steps, including rearrangement of the T-cell receptor (TCR) locus. After positive and negative selection, naïve T cells expressing a diverse population of TCRs are released from the thymus into the periphery, where they proliferate after an antigen or homeostatic stimulus and differentiate into specific T-cell subsets. However, in adult humans, the naïve CD4+ T-cell pool seems to be largely maintained by post-thymic T-cell proliferation; the release of T cells from the thymus may significantly contribute to T-cell replenishment under specific conditions—for example, during T-cell reconstitution after hematopoietic stem cell transplantation [25]. Thus, we hypothesize that decreased thymic output of CD4+ T cells contributes to lymphopenia and compromised immune defense in sepsis. To evaluate this hypothesis, we quantified CD4+ cells that were recently released by the thymus (RTEs) by measuring TRECs in CD4+ T cells and flow cytometry. TRECs are excision byproducts generated during T-cell development in the thymus. These episomal DNA fragments are enriched in RTEs, because they are not replicated during cell division and their frequency progressively decreases during peripheral T-cell proliferation and differentiation. Thus, TRECs can be used to estimate T-cell release from the thymus; however, alterations in peripheral T-cell proliferation during sepsis may affect TREC levels [26]. In fact, we found elevated proliferation of T cells in septic patients compared to controls, albeit at a very low level. Therefore, we also investigated RTEs by flow cytometry staining, using the cell surface marker CD31, which is highly expressed in RTEs. Because peripheral proliferation of RTEs due to antigenic priming or homeostatic signals results in CD31 downregulation, RTEs can be distinguished from their proliferative offspring by the presence of CD31 on naïve T cells. Interestingly, we found decreased TRECs; we also found a decreased relative and absolute number of CD31+CD4+ T cells but an increased relative number of CD31-CD4+ T cells in septic patients. This indicates that thymic output of RTEs is decreased and that subsequent peripheral proliferation of CD4+ T cells is increased in septic patients. We also determined the plasma levels of IL-7, which plays key roles in thymopoiesis and peripheral naïve T-cell survival and low-level proliferation; we found decreased IL-7 levels in septic patients. Interestingly, substitution with recombinant human IL-7 reversed the marked loss of CD4+ and CD8+ immune effector cells in septic patients [27]. However, the effect on thymic output was not evaluated in this study. Thus, decreased IL-7 levels in our investigation may contribute to decreased CD4+ T cell numbers, however, it remains to be investigated if the increased IL-15 levels can specifically promote CD8+ T cell number maintenance that we observed in our study.

Decreased thymic output has also been described in T-cell immunodeficiencies, HIV infection, aging, and autoimmune diseases [28]. In a mouse model of sepsis, thymic output of CD4+ RTEs was reduced to approximately 11% in septic mice at up to eight days after the induction of sepsis; this output quickly recovered subsequently [29]. Although the thymus plays a crucial role in establishing naïve T cells with a diverse TCR repertoire, TCR transcript diversity was not significantly different between control mice and septic mice in this study [29]. In contrast, a decreased TCR repertoire diversity was reported in peripheral blood mononuclear cells of sepsis patients [30,31], suggesting the functional relevance of decreased thymic output in human sepsis. Differences of TCR repertoire diversity may be due to the fact that mice maintain their naïve T cell pool throughout their lifetime through thymus output while thymus function decreases in adult humans and they maintain the naïve T cell pool through peripheral T cell division [32]. These issues may be particularly important in elderly septic patients with limited T cell repertoire [33]. Although the cause of decreased thymic output remains unclear, our findings are compatible with a very early report on sepsis-induced thymic atrophy [34].

Interestingly, we also found increased CD31-CD4+ T cells in sepsis, suggesting peripheral proliferation of naïve CD4+ T cells to substitute for T-cell depletion, a phenomenon known as lymphopenia-induced homeostatic proliferation (HP). However, it has been shown in an animal model of sepsis that, although naïve CD8+ T cells undergo HP, naïve CD4+ T cells fail to undergo HP. However, because there were high numbers of activated CD4+ T cells when the T-cell compartment had recovered after sepsis, other non-HP mechanisms have been suggested to explain CD4+ T-cell recovery [35]. Although peripheral proliferation may compensate for T cell numbers and improve immune function, replenishment of the naïve T-cell pool by high thymic output of RTEs is necessary for full immune recovery [36].

Increased peripheral proliferation may lead to replicative senescence. However, unlike other somatic cells, lymphocytes can upregulate telomerase upon activation, thereby maintaining telomere length despite extensive proliferation during infection [37]. In contrast, we found decreased telomere length in CD4+ T cells but not CD8+ T cells of septic patients, despite upregulated telomerase and moreover despite upregulated shelterin complexes, which protect telomeres from shortening. Similarly to our study, decreased telomere length was also observed in a mouse model of sepsis and in human peripheral blood cells of septic patients, which was explained as possibly resulting from oxidative stress or the release of several inflammatory factors [38]. In this case, increased telomerase activity cannot compensate for these mechanisms. However, we could not find differences in telomerase activity in CD4 and CD8+ T cells. Thus, it is purely speculative that CD8+ cells may be less prone to mechanisms of telomere shortening such as oxidative stress. IL-7 and IL-15 are involved in regulation of T cell senescence, in particular by upregulation of the telomerase [39]. Interestingly, it was shown previously that CD8+ T cells can maintain telomere length in presence of IL-15 [40].

This study has several limitations. The overall sample size of this observational study was relatively small, and all study participants were recruited from a single center. In addition, the time point of blood collection from sepsis patients was variable and occurred within the first 48–72 h after diagnosis of sepsis. Functional studies on the different T cell populations and TCR repertoire could provide further insight into T cell defects in future.

In conclusion, our investigation addresses, for the first time, the importance of thymic function for the maintenance of the naïve CD4+ T-cell compartment in septic patients; we have identified decreased thymic output as a possible confounder for septic lymphopenia and immune suppression. These findings are particularly relevant for lymphopenic clinical settings that require the appraisal of thymus-targeting strategies to reconstitute the T-cell pool, such as in the context of IL-7 substitution.
