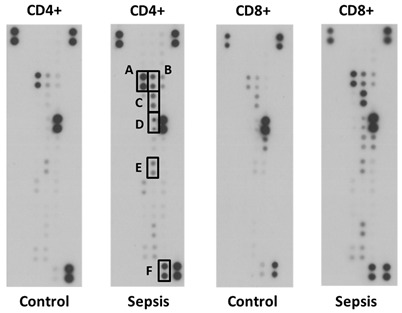# Correction: Sommer et al. Decreased Thymic Output Contributes to Immune Defects in Septic Patients. *J. Clin. Med.* 2020, *9*, 2695

**DOI:** 10.3390/jcm11154497

**Published:** 2022-08-02

**Authors:** Natascha Sommer, Steffen Noack, Andreas Hecker, Holger Hackstein, Gregor Bein, Norbert Weissmann, Werner Seeger, Konstantin Mayer, Matthias Hecker

**Affiliations:** 1 Excellence Cluster Cardio-Pulmonary Institute (CPI), University of Giessen and Marburg Lung Center (UGMLC), Member of the German Center for Lung Research (DZL), Justus-Liebig-University of Giessen, 35392 Giessen, Germany; natascha.sommer@innere.med.uni-giessen.de (N.S.); steffen_noack@gmx.de (S.N.); norbert.weissmann@innere.med.uni-giessen.de (N.W.); werner.seeger@innere.med.uni-giessen.de (W.S.); konstantin.mayer@innere.med.uni-giessen.de (K.M.); 2 Department of General and Thoracic Surgery, Justus-Liebig-University of Giessen, 35392 Giessen, Germany; andreas.hecker@chiru.med.uni-giessen.de; 3 Department of Transfusion Medicine and Hemostaseology, University Hospital Erlangen, 91054 Erlangen, Germany; holger.hackstein@uk-erlangen.de; 4 Institute for Clinical Immunology and Transfusion Medicine, Justus-Liebig-University, 35392 Giessen, Germany; gregor.bein@immunologie.med.uni-giessen.de; 5 Department of Pulmonary and Sleep Medicine, ViDia Hospitals, 76137 Karlsruhe, Germany

In the original article, there were errors in Figure 1D (panel CD8-Control) [1]. Changes are below. The authors apologize for any inconvenience caused and state that the scientific conclusions are unaffected. The original article has been updated.

From


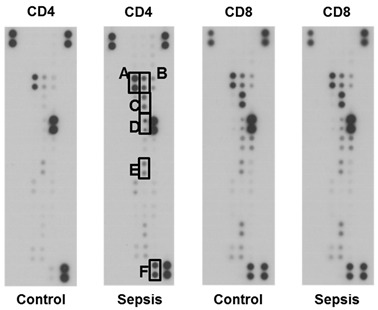


to